# Supplementary material for: Automatic Counterfeit Currency Detection Using a Novel Snapshot Hyperspectral Imaging Algorithm
Source: Sensors (Basel). 2023 Feb 10;23(4):2026. doi: 10.3390/s23042026 (PMC9967082; doi:10.3390/s23042026)
Supplement: Supplementary file 1 [file sensors-23-02026-s001.zip › sensors-2182037-supplementary.pdf]

sensors

MDPI

Supplementary Material

Automatic Counterfeit Currency Detection Using a Novel Snapshot Hyperspectral Imaging Algorithm

Arvind Mukundan [1], Yu-Ming Tsao [1], Wen-Min Cheng [2,3], Fen-Chi Lin [4,*] and Hsiang-Chen Wang [1,5,*]

1 Department of Mechanical Engineering, Advanced Institute of Manufacturing with High tech Innovations (AIM-HI) and Center for Innovative Research on Aging Society (CIRAS), National Chung Cheng University, 168, University Rd., Min Hsiung, Chia Yi 62102, Taiwan
2 College of Management, National Sun Yat-sen University, 70 Lienhai Rd., 80424 Kaohsiung, Taiwan
3 Medical Devices R&D Service Development, Metal Industries Research & Development Centre, 1001 Kaonan Highway, Nanzi Dist., Kaohsiung, Taiwan
4 Ophthalmology, Kaohsiung Armed Forces General Hospital, 2, Zhongzheng 1st.Rd., Lingya District, Kaohsiung City 80284, Taiwan
5 Director of Technology Development, Hitspectra Intelligent Technology Co., Ltd., 4F, No.2, Fuxing 4th Rd., Qianzhen District, Kaohsiung 80661, Taiwan
* Correspondence: eses.taiwan@gmail.com (F.-C.L.); hcwang@ccu.edu.tw (H.-C.W.)

1. Instrument Specification

In this study, a total of six instruments were used, including a microprocessor, monocular camera, LED strip, diffuser, LED dimmer, and TFT touch screen. Table S1 describes all the instruments used. The microprocessor used was a Raspberry Pi 3 Model B+, which has 1GB LPDDR2 SDRAM. It was operated using a computer through VLC. The camera used to capture the hologram was a Raspberry Pi Camera Module 2 which has a Sony IMX219 8-megapixel sensor. The screen used to control the processor was Adafruit's 2.8-inch PiTFT Touchscreen. A 3000K COB light was also used, along with a dimmer. An opal white diffuser sheet was used to diffuse the light evenly onto the hologram.

Table S1. Components used to build the module.

Part	Component	Model	Cost	Manufacturer
Processor	Raspberry Pi V3	Model B+	$35.00	Raspberry Pi
Camera	Raspberry Pi Camera	V2	$25.00	Raspberry Pi
LED and Dimmer	COB LED Strip	COB	$25.00	PAUTIX
Diffuser	Opal Diffuser	-	$5	Guangzhou co. ltd
TFT Screen	320x240 2.8" TFT	-	$35	Adafruit

2. Entropy Measurement

In this study, the entropy of the two duplicate and two original banknotes was selected and measured. Entropy in an image can be defined as the measure of the degree of randomness in the image. Figures S1–S7 represent the entropy of the first region of interest (ROI). These figures suggest that the entropy of all samples is distinctively different. Therefore, differentiating these samples using MGVs was easy. Entropy is used to measure the randomness of the image. The original has more characteristics in this specific ROI than the duplicate currency. Hence, the entropy of the original currency is more than the duplicate currency.

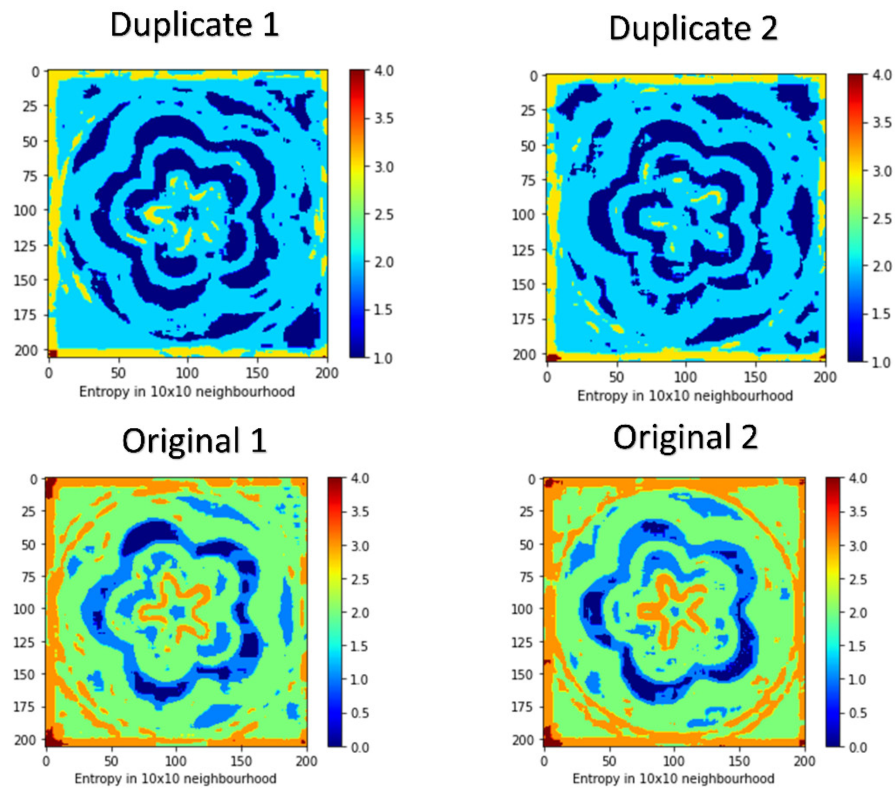

Figure S1. Entropy of two samples of original and duplicate in the ROI 1 at the wavelength of 400 nm. Entropy of Duplicate sample 1 (Top left corner), Entropy of Duplicate sample 2 (Top right corner), Entropy of Original sample 1 (Bottom left corner), Entropy of Original sample 2 (Bottom right corner).

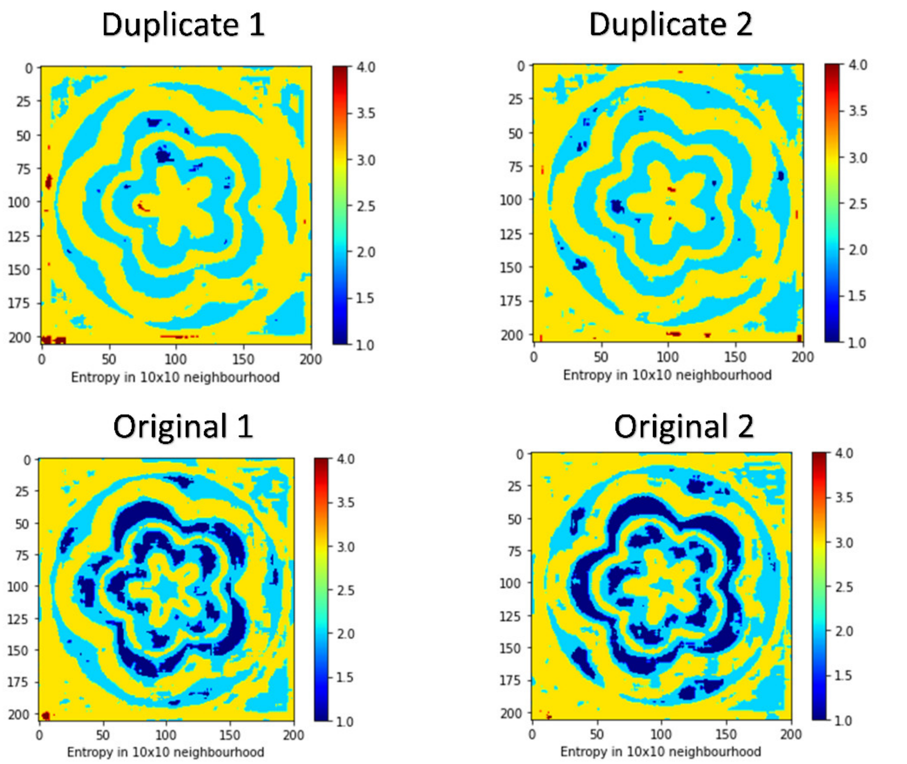

Figure S2. Entropy of two samples of original and duplicate in the ROI 1 at the wavelength of 450 nm. Entropy of Duplicate sample 1 (Top left corner), Entropy of Duplicate sample 2 (Top right

corner), Entropy of Original sample 1 (Bottom left corner), Entropy of Original sample 2 (Bottom right corner).

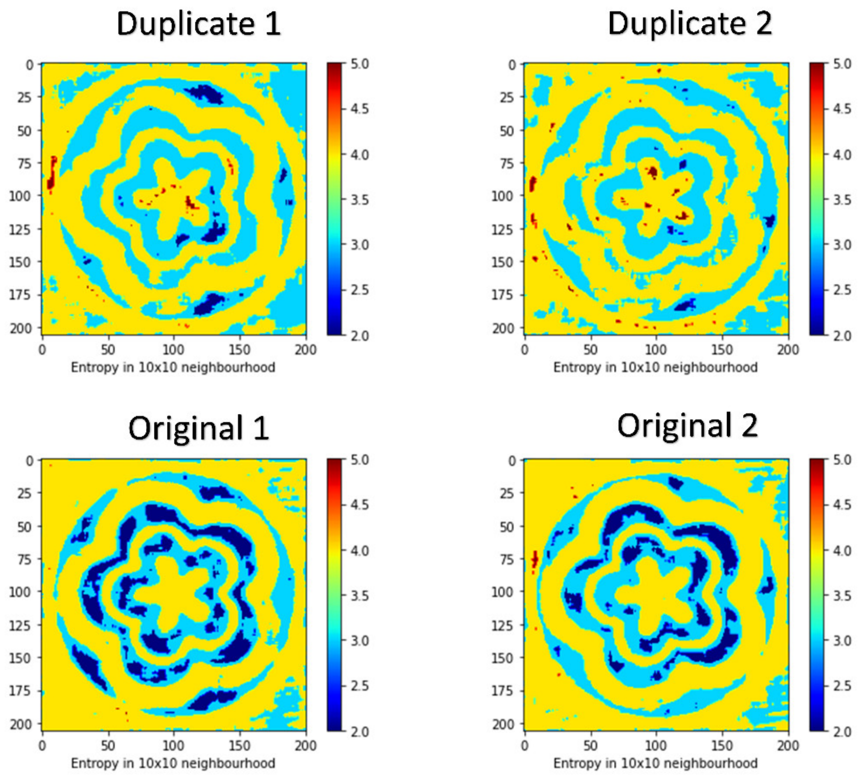

Figure S3 Entropy of two samples of original and duplicate in the ROI 1 at the wavelength of 500 nm. Entropy of Duplicate sample 1 (Top left corner), Entropy of Duplicate sample 2 (Top right corner), Entropy of Original sample 1 (Bottom left corner), Entropy of Original sample 2 (Bottom right corner).

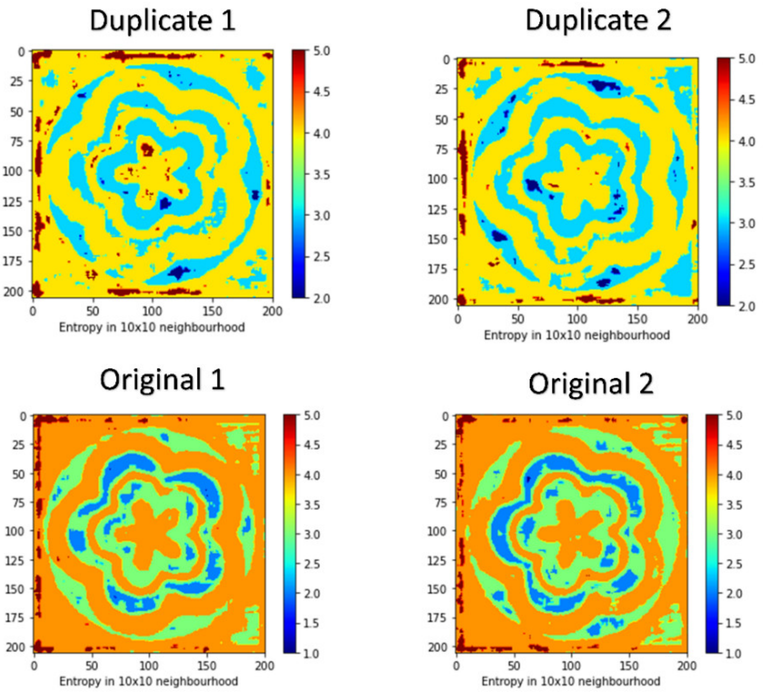

Figure S4. Entropy of two samples of original and duplicate in the ROI 1 at the wavelength of 550 nm. Entropy of Duplicate sample 1 (Top left corner), Entropy of Duplicate sample 2 (Top right

corner), Entropy of Original sample 1 (Bottom left corner), Entropy of Original sample 2 (Bottom right corner).

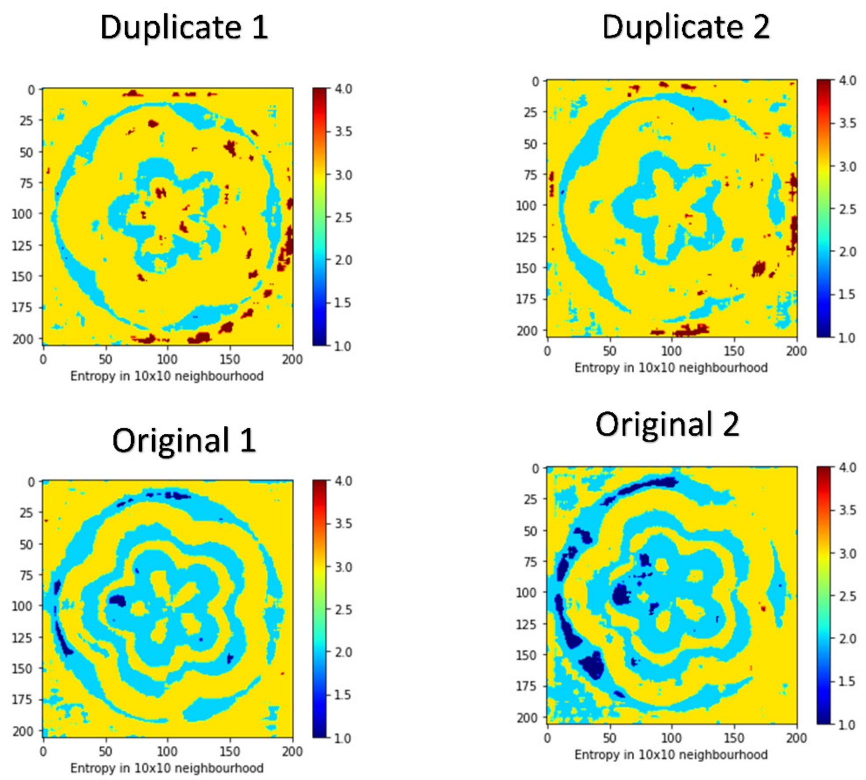

Figure S5. Entropy of two samples of original and duplicate in the ROI 1 at the wavelength of 600 nm. Entropy of Duplicate sample 1 (Top left corner), Entropy of Duplicate sample 2 (Top right corner), Entropy of Original sample 1 (Bottom left corner), Entropy of Original sample 2 (Bottom right corner).

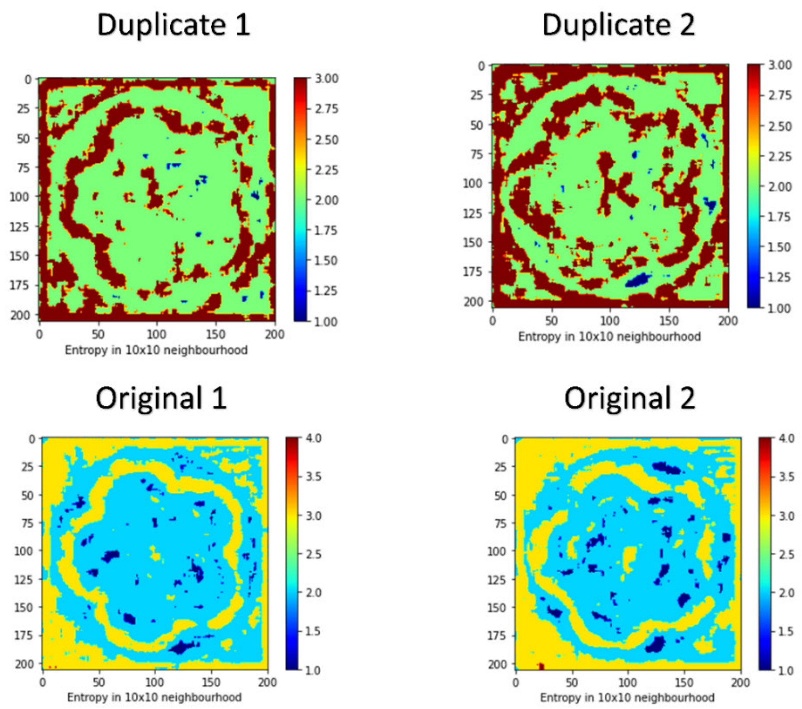

Figure S6. Entropy of two samples of original and duplicate in the ROI 1 at the wavelength of 650 nm. Entropy of Duplicate sample 1 (Top left corner), Entropy of Duplicate sample 2 (Top right

corner), Entropy of Original sample 1 (Bottom left corner), Entropy of Original sample 2 (Bottom right corner).

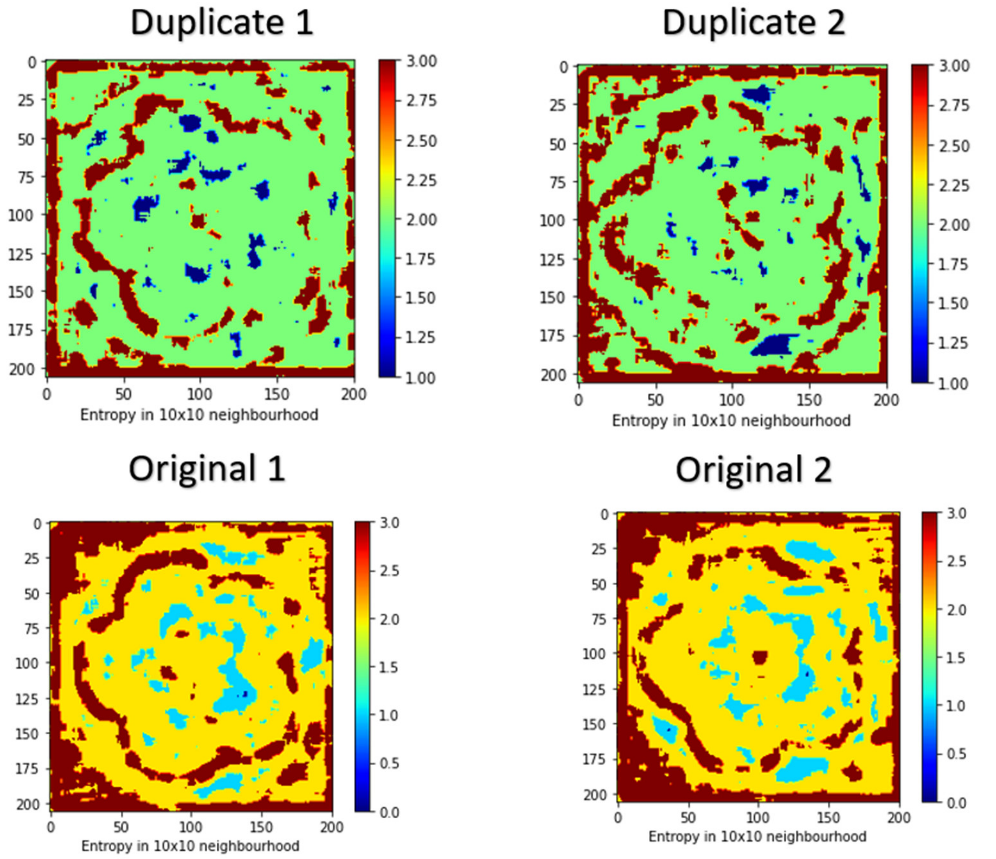

Figure S7. Entropy of two samples of original and duplicate in the ROI 1 at the wavelength of 700 nm. Entropy of Duplicate sample 1 (Top left corner), Entropy of Duplicate sample 2 (Top right corner), Entropy of Original sample 1 (Bottom left corner), Entropy of Original sample 2 (Bottom right corner).

3. Visible Hyperspectral Imaging Algorithm

The visible hyperspectral imaging (VIS-HSI) used in this study is calculated by using the images taken by a single-lens camera (Nikon D5200) combined with the visible hyperspectral algorithm (VIS-HSA). The wavelength range is from 400 nm to 700 nm, and the spectral resolution is up to 1 nm. The detailed instrument specifications used in this study are mentioned in Table S2.

Table S2. Instrument specification.

	Specification	Resolution	Components	Bilateral
Visible Light Camera	Raspberry Pi camera	6000*4000	CMOS	400–700nm
Spectrometer	Ocean Optics QE65000	1mm	CCD	200–1100nm

The individual conversion formulas to convert the 24-color patch image and 24-color patch reflectance spectrum data to *XYZ* color space are as follows.

On the camera side: convert sRGB color gamut space to *XYZ* color gamut space.

$$\begin{bmatrix} X \\ Y \\ Z \end{bmatrix} = [M_A][T] \begin{bmatrix} f(R_{sRGB}) \\ f(G_{sRGB}) \\ f(B_{sRGB}) \end{bmatrix} \times 100\,, 0 \le \begin{matrix} R_{sRGB} \\ G_{sRGB} \\ B_{sRGB} \end{matrix} \le 1 \quad \text{(S1)}$$

$$[T] = \begin{bmatrix} 0.4104\ 0.3576\ 0.1805 \\ 0.2126\ 0.7152\ 0.0722 \\ 0.0193\ 0.1192\ 0.9505 \end{bmatrix} \quad \text{(S2)}$$

$$f(n) = \begin{cases} (\frac{n+0.055}{1.055})^{2.4}, n > 0.04045 \\ \left(\frac{n}{12.92}\right), otherwise \end{cases} \quad \text{(S3)}$$

$$[M_A] = \begin{bmatrix} X_{SW}/X_{CW} & 0 & 0 \\ 0 & Y_{SW}/Y_{CW} & 0 \\ 0 & 0 & Z_{SW}/Z_{CW} \end{bmatrix} \quad \text{(S4)}$$

On the spectrometer side: convert reflection spectral data to *XYZ* color gamut space.

$$X = k\int_{400nm}^{700nm} S(\lambda)R(\lambda)\bar{x}(\lambda)d\lambda \quad \text{(S5)}$$

$$Y = k\int_{400nm}^{700nm} S(\lambda)R(\lambda)\bar{y}(\lambda)d\lambda \quad \text{(S6)}$$

$$Z = k\int_{400nm}^{700nm} S(\lambda)R(\lambda)\bar{z}(\lambda)d\lambda \quad \text{(S7)}$$

$$k = 100/\int_{400nm}^{700nm} S(\lambda)\bar{y}(\lambda)d\lambda \quad \text{(S8)}$$

The nonlinear response of the camera can be corrected by a third-order equation, and the nonlinear response correction variable is defined as $V_{Non\text{-}linear}$.

$$V_{Non-linear} = [X^3\ Y^3\ Z^3\ X^2\ Y^2\ Y^2\ X\ Y\ Z\ 1]^T \quad \text{(S9)}$$

In the dark current part of the camera, the dark current is usually a fixed value and does not change with the amount of incoming light, so a constant is given as the contribution of the dark current, and the dark current correction variable is defined as V_{Dark}.

$$V_{Dark} = [a] \quad \text{(S10)}$$

Finally, V_{Color} is used as the base, and multiplied by the nonlinear response correction of $V_{Non\text{-}linear}$; the result is standardized within the third order to avoid excessive correction, and finally, V_{Dark} is added to obtain the variable matrix V.

$$V_{Color} = [XYZ\ XY\ XZ\ YZ\ X\ Y\ Z]^T \quad \text{(S11)}$$

$$V = [X^3\ Y^3\ Z^3\ X^2Y\ X^2Z\ Y^2Z\ XY^2\ XZ^2\ YZ^2\ XYZ\ X^2\ Y^2\ Y^2\ XY\ XZ\ YZ\ X\ Y\ Z\ a]^T \quad \text{(S12)}$$

Before using CIE DE2000 to calculate color difference, $XYZ_{Correct}$ and $XYZ_{Spectrum}$ must be converted from XYZ color space to lab color space. The conversion formula is as follows:

$$L^* = 116f\left(\frac{Y}{Y_n}\right) - 16$$
$$a^* = 500\left[f\left(\frac{X}{X_n}\right) - f\left(\frac{Y}{Y_n}\right)\right] \quad \text{(S13)}$$
$$b^* = 200\left[f\left(\frac{Y}{Y_n}\right) - f\left(\frac{Z}{Z_n}\right)\right]$$

$$f(n) = \begin{cases} n^{\frac{1}{3}}, & n > 0.008856 \\ 7.787n + 0.137931, & otherwise \end{cases} \quad \text{(S14)}$$

4. Spectral Signature

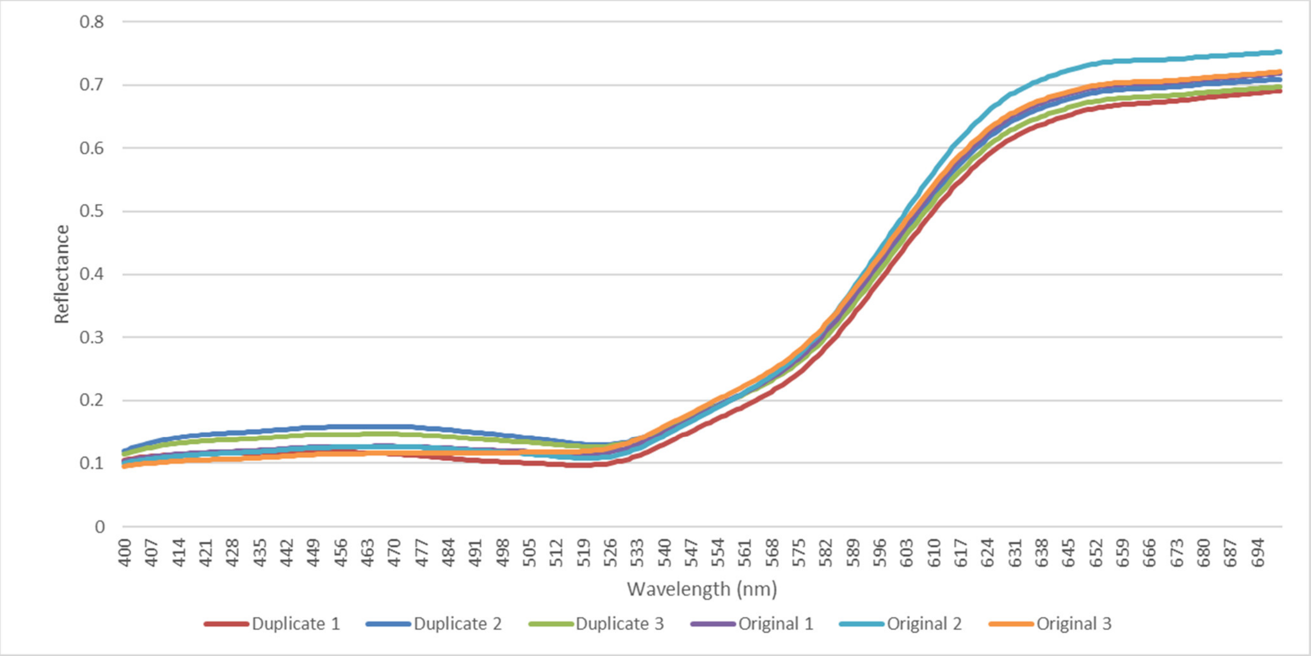

Figure S8. Spectral reflectance of the three original and three duplicate banknotes between 400 nm and 700 nm in ROI 1.

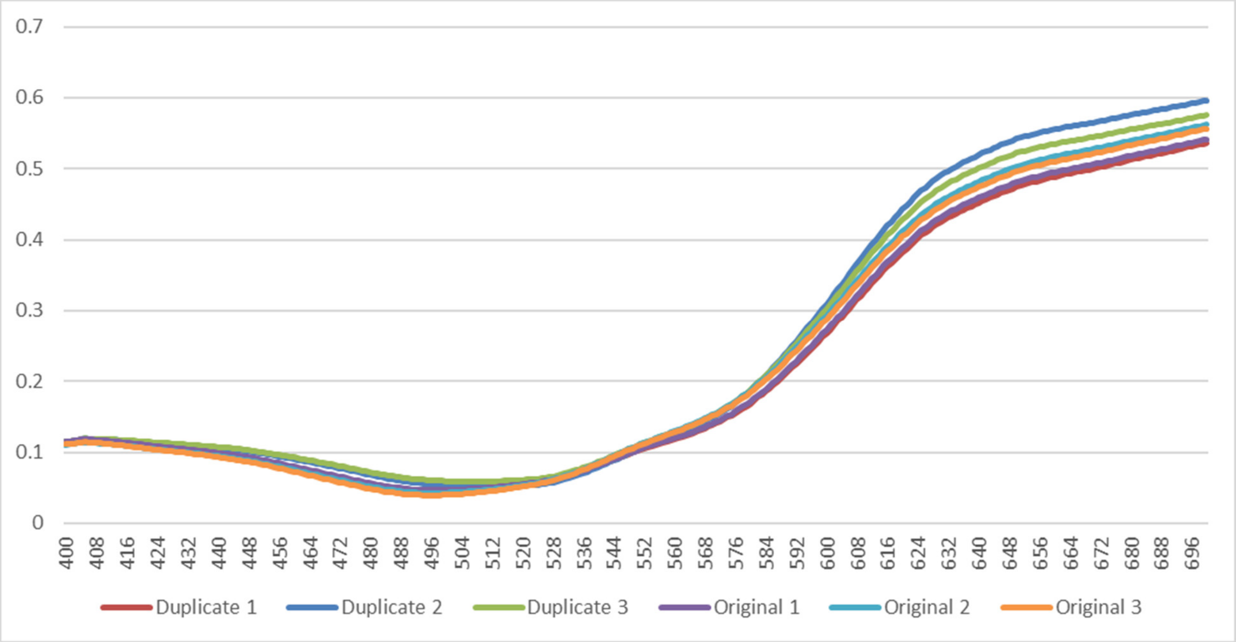

Figure S9. Spectral reflectance of the three original and three duplicate banknotes between 400 nm and 700 nm in ROI 2.

5. Mean Gray Value

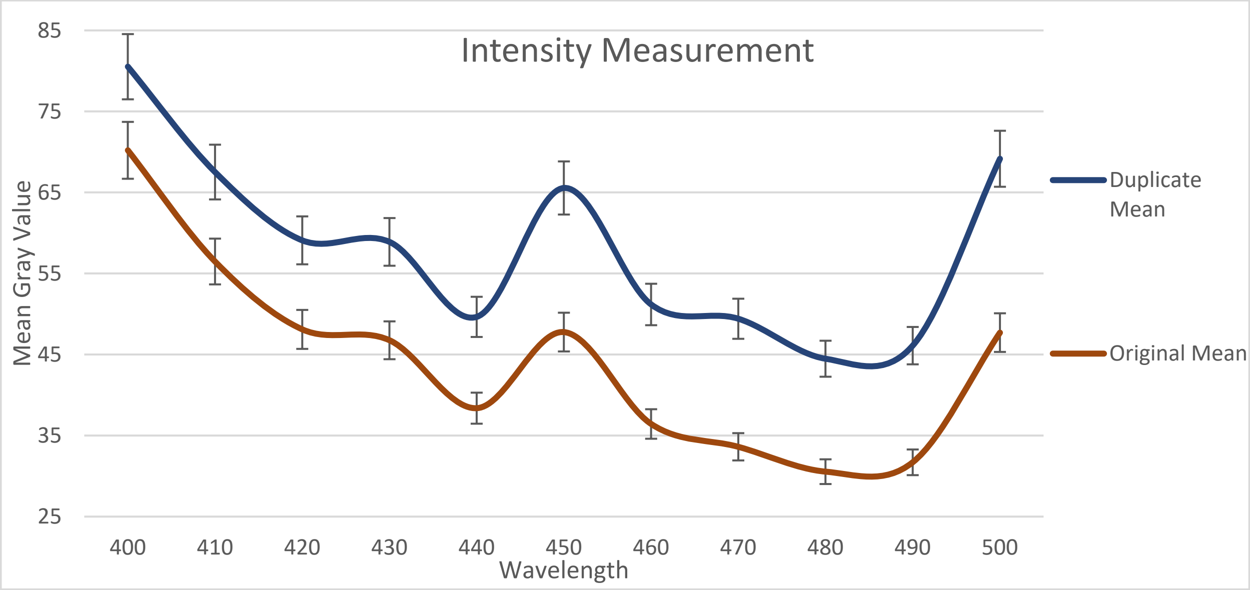

Figure S10. Mean of the duplicate and original MGVs of the original and duplicate samples in ROI 1.

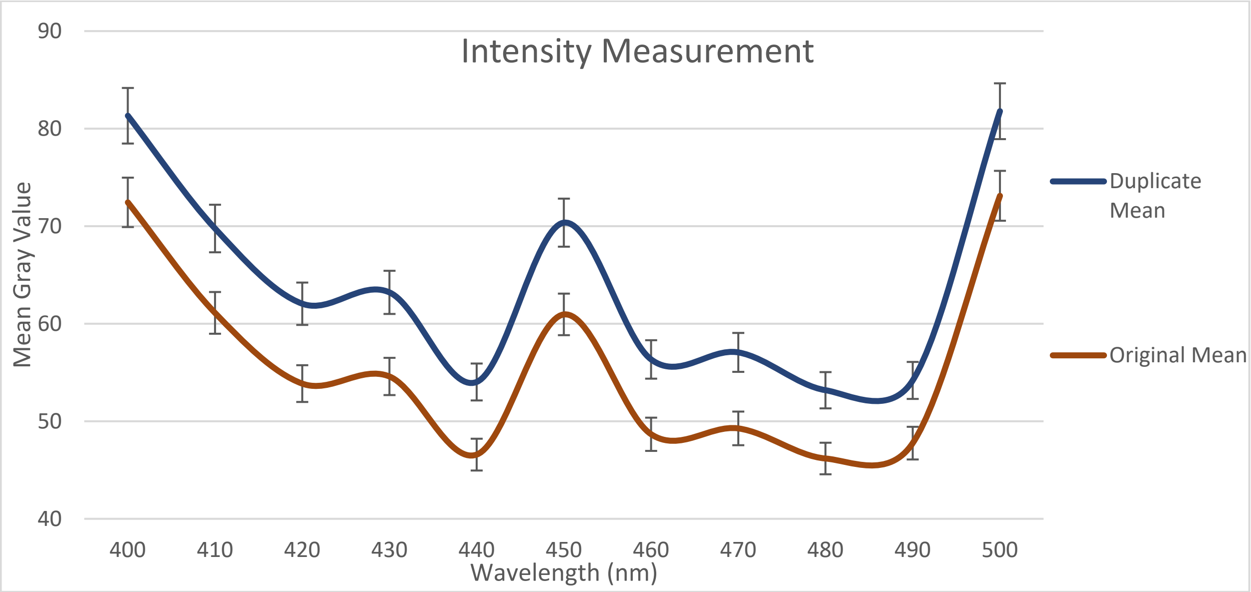

Figure S11. Mean of the duplicate and original MGVs of the original and duplicate samples in ROI 2.

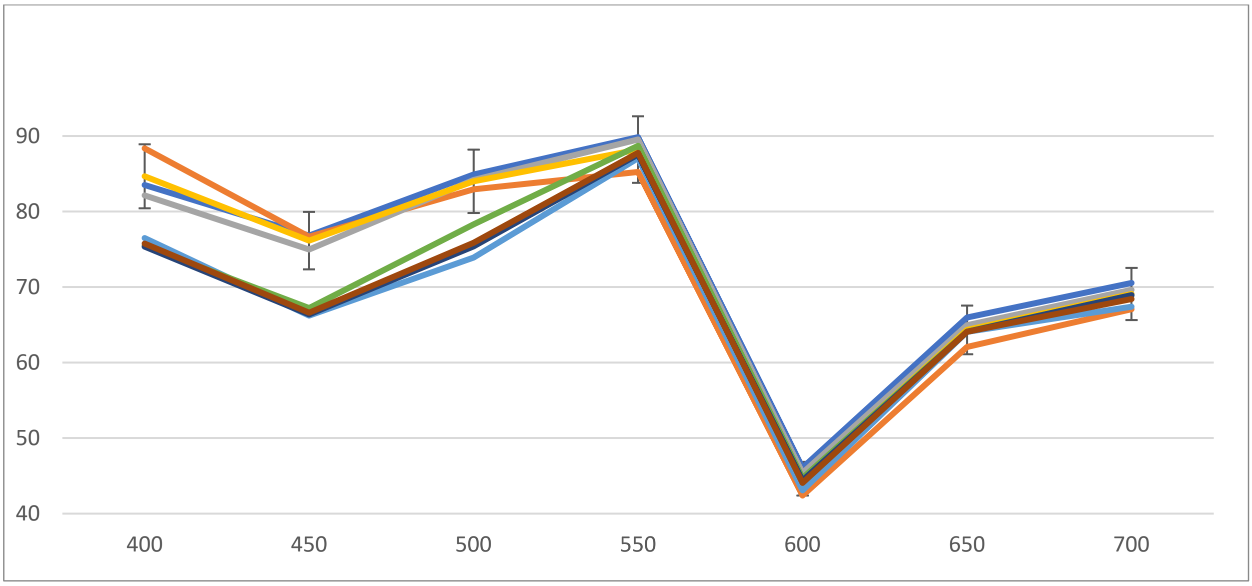

Figure S12. Mean of the duplicate and original MGVs of the original and duplicate samples in ROI 1.

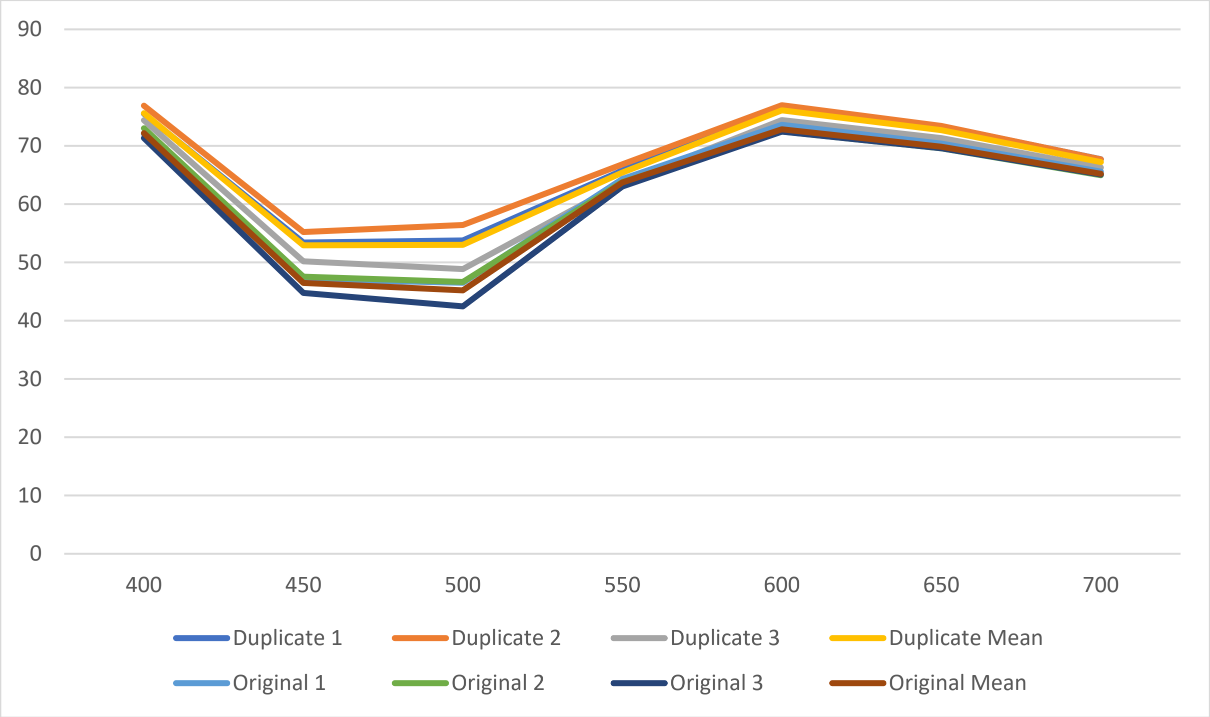

Figure S13. Mean of the duplicate and original MGVs of the original and duplicate samples in ROI 2.

6. MGV of the RGB image

Table S3. MGVs of the RGB image.

	**Duplicate 1**	**Duplicate 2**	**Duplicate 3**	**Duplicate Mean**	**Original 1**	**Original 2**	**Original 3**	**Original Mean**
ROI 1	71.95136	71.10605	71.96940	71.67560	68.3129	69.6284	68.8642	68.93522
ROI 2	63.63156	64.63540	64.15943	64.14213	62.7878	62.5742	61.2455	62.20254
